# The Relationship between Power Type, Work Engagement, and Organizational Citizenship Behaviors

**DOI:** 10.3390/ijerph16061015

**Published:** 2019-03-20

**Authors:** Kwang O. Park

**Affiliations:** Division of Business, Yeungnam University College, 170 Hyeonchung-ro, Nam-gu, Daegu 42415, Korea; kopark1021@ync.ac.kr; Tel.: +82-53-650-9333; Fax: +82-53-625-6246

**Keywords:** coercive power, non-coercive power, work engagement, organizational citizenship behavior

## Abstract

The focus of this study is to investigate if power type improves organizational citizenship behavior (OCB) through work engagement. Based on existing research, power can be classified into two main types: coercive and non-coercive power. Coercive power is divided into the categories of coercion, reward, and legitimate power, and non-coercive power can be divided into information, expert, and reference power. Therefore, this study examines what kind of relationship is formed in the work engagement of organization members based on power type, and ultimately empirically investigates the effects on OCB. Although it is very important in organizational research, no study has yet been conducted on the relationships between power type, work engagement, and OCB. The survey targets of this study were the companies listed on the Korea Composite Stock Price Index (KOSPI), a stock market in South Korea. The companies listed on the KOSPI are the representative companies of South Korea, as announced by the South Korean government based on their market representativeness, liquidity, and industry representativeness. This study sheds new light on the relationships between power type, work engagement, and OCB which have been overlooked from both the academic and practical perspectives. Based on this study, it is expected that power types that have practical influence will be further investigated, and the plans required for the maintenance of better relationships in an organization could then be established.

## 1. Introduction

To secure a continuous competitive edge, a company needs talented people. An effective and motivated human resource (HR) is an essential soft power which competitors cannot easily imitate. In other words, as far as the maintenance of competitive edge is concerned, the social, political, and cultural aspects of an organization are more important than the technical aspects, and based on this, it is essential to improve the dedication and involvement of the members of the organization.

Power is a critical element in an organization. It is defined as the ability to influence the decision-making, intention, and behavior of a company [1]. Power is mainly classified into two overarching types: coercive power (coercion, reward, legitimate) and non-coercive power (information, expert, reference) [2]. Ever since Hunt and Nevin [3] defined power, many researchers have conducted empirical studies by classifying it more diversely.

Depending on the power type in an organization, the involvement and loyalty of organization members can vary [4]. Nowadays, organizations seek members who produce creative outcomes by changing their roles and giving their energy as and when needed, rather than those who simply deal with only their given tasks. Up until now, however, the methods selected by a majority of companies to motivate the members have primarily focused on the elimination of dissatisfaction or improvement of satisfaction of the members through evaluation and compensation system improvement. Satisfaction of members has a large effect on organization performance [5].

Among the constructs of motivation theory, work engagement is the last topic discussed. According to Rich et al. [6], work engagement is defined as job involvement, work satisfaction, and intrinsic motivation, and has a larger effect on work performance than other variables. Moreover, it is a complex construct encompassing all the physical, emotional, and cognitive aspects of individual persons.

As such, work engagement, whereby members are completely involved in their own roles through intrinsic motivation, is an important topic of discussion currently in HR. Furthermore, companies are recognizing the work engagement of members as an important driver that increases the company’s competitiveness. Work engagement is related to the performance of members, success of the organization and the financial performance of the company, including shareholder earnings [7].

Gallup [8] reported that, in the case of the United States, the losses incurred due to poor productivity of workers with low work engagement amounts to $300 billion annually. Furthermore, only 29% of US workers reported a state of high work engagement, while 54% of workers reported an average level, and 17% of workers reported being in a disengaged state.

At present, another important issue for companies is organization citizenship behavior (OCB). OCB includes the tasks that are not the official responsibilities of members, but rather are voluntary and functional actions performed by them to contribute to the performance of the organization. This refers to extra-role behaviors that contribute to the accomplishment of organizational goals, by enabling the smooth running of the social functions of an organization and reducing friction between members [9]. OCB in an organization is important because it has an effect on the organization’s performance [10].

There is no doubt that the survival and advancement of an organization depends on the behaviors of members. However, even if members work hard on their given tasks, the organization’s performance or organizational effectiveness will not necessarily improve. For the survival and advancement of an organization, certain types of members are particularly important: those who look for and perform tasks on their own, even if it is not their work officially, and those who take initiative without expecting any reward for their work. To improve the effectiveness of an organization, not only the in-role behaviors of members but also OCB, including extra-role behaviors, are very important.

**Figure 1:**
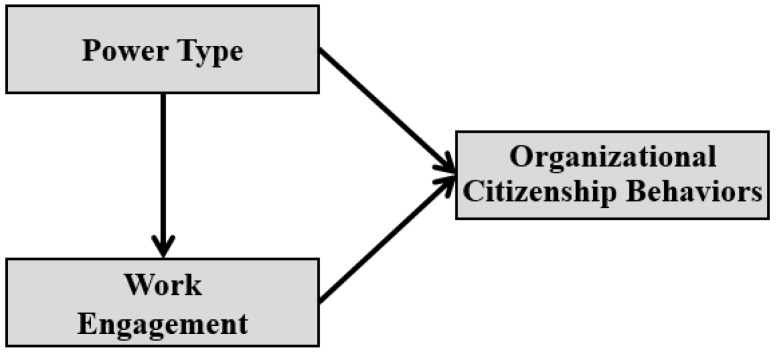
Conceptual research model.

Therefore, the focus of this study is to investigate if power type influences OCB through work engagement. Based on the studies of Hausman and Johnston [11] and Benton and Maloni [12], we classified power into two types: coercive and non-coercive power. Coercive power was further divided into coercion, reward, and legitimate power, and non-coercive power was divided into information, expert, and reference power [13]. Therefore, this study examines what kind of relationship is formed in terms of the work engagement of organization members based on power type, and ultimately empirically investigates the effects on OCB. For this purpose we formed a conceptual research model, as shown in Figure 1, and attempted to answer the following two research questions:

**Question** **1:** Does the power type have an influence on work engagement?

**Question** **2:** Does work engagement have an influence on OCB?

To answer these two questions, this study targeted companies listed on the Korea Composite Stock Price Index (KOSPI), which is a stock market in South Korea. These companies are the representative companies of South Korea, announced by the South Korean government based on their market representativeness, liquidity, and industry representativeness.

Traditionally, a majority of business studies focus on US-based companies. This is because the USA plays a leading role in many social and business areas. However, studies with a global perspective are also necessary. Owing to the advancement of information technology (IT) and the Internet, some countries have advanced as much as the USA in certain areas. In an index announced by the Economist Intelligence Unit (EIU) of the United Kingdom (U.K.), South Korea ranked number three after the USA and Japan. According to Robert [14] of the U.K., when measuring the IT human resource index for 66 countries around the world, South Korea scored 58.9 points, ranking number two behind the USA on 75.6 points. Furthermore, South Korea ranked number six in the IT competitiveness index. Therefore, in a gradually globalizing market, studies should be conducted with international perspectives. Moreover, because companies conduct business multinationally at present, studies of more comprehensive perspectives are required.

Accordingly, our study is expected to contribute to finding an effective performance accomplishment method, from both the practical and theoretical perspectives, for companies striving to strengthen their competitive advantages.

## 2. Theoretical Review and Hypothesis Development

**Figure 2:**
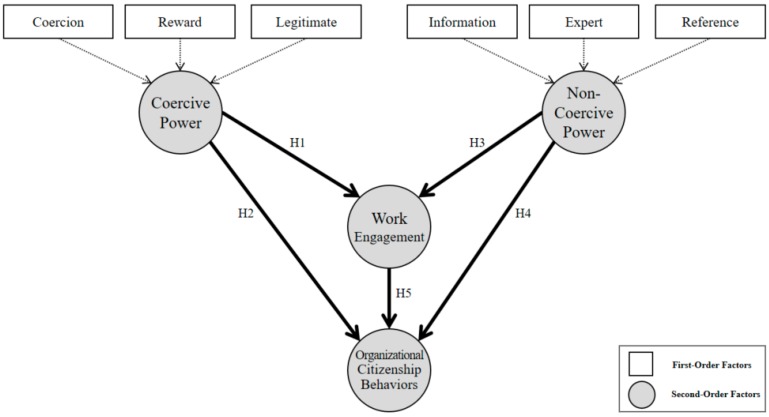
Research model, H1–H5: Hypothesis 1–5.

This study examines the causal relationships between coercive power/non-coercive power and work engagement, and ultimately aims to analyze the effect on OCB empirically, through a structural equation model. Therefore, the concepts constructed in the research model of this paper were designed as shown in Figure 1, and the research hypotheses were derived as shown in Figure 2.

### 2.1. Hypothesis Development

**Figure 3:**
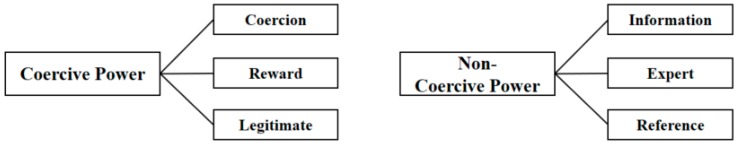
Power type.

Power is can be classified into coercive power (coercion, reward, legitimate, shown in Figure 3) and non-coercive power (information, expert, reference) [11,12]. Table 1 is an example of a list of the existing research on power.

Coercion, reward, and legitimate power, which are classified under coercive power, occur when achievement of a certain goal is difficult because the maintenance of a favorable relationship with a counterpart is impossible. By coercing the behaviors of counterparts and exercising binding punishment and legitimate power, these powers are created in unavoidable situations that are accompanied by the obedience of counterparts [12].

Coercive power decreases the involvement of workers. Furthermore, the coercive behavior of a superior has a negative effect on the work engagement of the organization’s members [7]. This ultimately induces negative emotions and decreases loyalty [12]. Therefore, even when some economic benefits are provided based on a short-term reward power, the use of coercive power lowers the satisfaction of workers, thereby decreasing their pride in the company long-term [13]. Based on the above, it is hypothesized that coercive power, including coercion, reward, and legitimate power, will have an influence on work engagement and OCB.

**Hypothesis** **1:** Coercive power will have influence on work engagement.

**Hypothesis** **2:** Coercive power will have influence on OCB.

Information, expert, and reference power, which are classified under non-coercive power, occur because of the attractiveness or personal magnetism of the counterparts through their exemplary work performance [17,18,19]. In general, the influence of this type of power is large when there is a belief that the culture or value of a counterpart is amicable and attractive [20,21,22]. As such, non-coercive power is an important factor in decision-making and impacting the behavior of a counterpart [23,24,25]. Furthermore, the expertise and reference of a superior increases the work engagement of organizational members [26,27]. In a meta-analysis of OCB, Ilies et al. [28] revealed that when the organization members have a good relationship with their superiors, there is a higher chance of better performance by the members because of the additional support, information, advice, and opportunities provided by the superiors. Based on the above, hypotheses are established that non-coercive power, such as information, expert, and reference power, will influence work engagement and OCB.

**Hypothesis** **3:** Non-coercive power will have an influence on work engagement.

**Hypothesis** **4:** Non-coercive power will have an influence on OCB.

It is emphasized that an individual with high work engagement will willingly use his/her passion and energy for OCB, in addition to performing his/her own role [24,29]. Because work engagement expands the range of activities that are considered to be the role of the individual, members with high work engagement do not discriminate between self and others when working [30,31]. When studying the relationship between work engagement and OCB, work engagement has been shown to have a positive effect on OCB [6]. Based on the above, a hypothesis is established that work engagement will influence OCB.

**Hypothesis** **5:** Work engagement will have an influence on OCB.

### 2.2. Sample and Data Collection

To verify the model and hypotheses of this study, an analysis was performed through a survey created based on conventional studies. The questionnaire was specifically designed for this study based on the references shown in Table 2. To conduct a more systematic survey, a pilot test was carried out by revealing the purpose of this study, and sending the survey to the human resource manager of a participating company. The survey targets of this study were the companies listed on the KOSPI, a stock market in South Korea. The companies listed on the KOSPI are the representative companies of South Korea, as announced by the South Korean government based on their market representativeness, liquidity, and industry representativeness. A total of 250 copies of the survey were distributed through phone, e-mail, and direct visit from May to July 2018. The number of responses received was 191, with about a 76% recovery rate. From among these, those with missing values were excluded and, finally, data of 184 persons were used for the final research analysis, as shown in Table 3. The statistical analysis was performed using SPSS 24.0 (IBM, Armonk, NY, USA) and AMOS 24.0 (IBM, Armonk, NY, USA).

As both independent and dependent variables were measured from the same respondents through the questionnaire, common method bias may exist. To verify this, we conducted Harmon’s one-factor test [31]. Through a factor analysis of the variables used in this study, we found that the factor explaining the largest amount of variance was 15.6%. In addition, as the variance of the sample explained by all factors was 75.4%, there is no problem of common method bias.

### 2.3. Measurement Model

In this study, a confirmatory factor analysis was performed to assess validity. To conduct the analysis, the number of factors was determined based on categories with an eigenvalue of 1 or higher, and the varimax rotation method was selected. The sample size required for a certain significance varies greatly, and if the sample size is 100 or higher, the factor loading required for the significance is 0.50–0.55 [32]. Therefore, in this study, the sample size was 184 factors, which were extracted based on a factor loading of 0.50 for the significance of factors. In the factor analysis results, all the factor loadings of coercion, reward, legitimate, information, expert, and reference power, work engagement, and OCB were higher than 0.50, as shown in Table 4. Therefore, it was determined that there is discriminant validity between the measured variables and convergent validity in the variables.

Furthermore, to measure the coercive power formed through coercion, reward, and legitimate power, and the non-coercive power formed through information, expert, and reference power, this study verified the research model through a second-order construct model. Moreover, this study used a structural equation model to verify the significance for the research hypotheses. Therefore, the convergent validity and discriminant validity were analyzed for the fitness evaluation of the research model. Convergent validity refers to the degree to which two or more measures of constructs are correlated, and it is determined using the construct reliability (CR) and average variance extracted (AVE). A CR value of 0.7 or higher and AVE of 0.5 or higher indicates the presence of a convergent validity [32]. As shown in Table 5, the CR values exceed the baseline 0.70, and the AVE values exceed the baseline 0.50. Thus, convergent validity is confirmed.

Discriminant validity refers to how much a construct is actually different from another construct. For two constructs which are targets of discriminant validity evaluation, the square root of AVE of each construct and the correlation of the two are compared to check if the square root of AVE is larger than the correlation. As shown in Table 6, since the square root of AVE is larger than the correlation between all the constructs, discriminant validity exists between all the constructs.

Furthermore, since the absolute value of the correlation coefficient of all the constructs does not exceed the baseline of 0.85, there is no multicollinearity problem between the constructs. Therefore, it can be seen that discriminant validity exists in the constructs overall [32]. Additionally, as shown in Table 7, the variance inflation factor (VIF) and tolerance (TOL) methods were used to examine the multicollinearity problem. The results of the analysis show that there is no multicollinearity problem between the variables. In general, when the VIF value is 10 or less and the TOL value is 0.3 or higher, it is considered that there is no multicollinearity problem.

We conducted structural equation analysis using AMOS 24. The fit statistics of this study were good, except for the CFI, as shown in Table 8 (*X*^2^/DF = 2.670, GFI = 0.912, RMSR = 0.041, RMSEA = 0.039, AGFI = 0.813, CFI = 0.892, TLI = 0.918, PGFI = 0.634) (Comparative Fit Index (CFI), Degrees of Freedom (DF), Goodness of Fit Index (GFI), Root Mean Square Residual (RMSR), Root Mean Square Error of Approximation (RMSEA), Adjusted Goodness of Fit Index (AGFI), Turker-Lewis Index (TLI), Parsimony Goodness of Fit Index (PGFI). As suggested by the index, it was judged to be acceptable to proceed with the analysis under the current conditions [33].

## 3. Results

**Figure 4:**
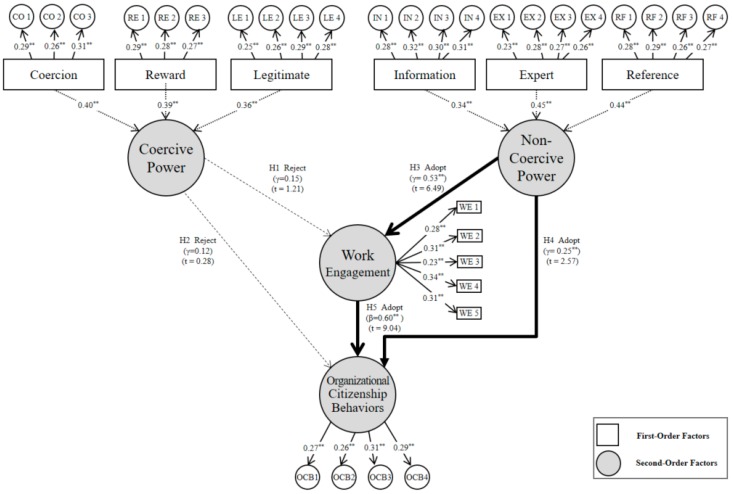
Results of hypothesis testing (* Significant at α = 0.05; ** significant at α = 0.01).

The results of the analysis are presented in Figure 4, and summarized in Table 9.

The hypothesis that coercive power influences work engagement (H1) was not statistically significant (*γ* = 0.15, *t* = 1.21). This result is different from the research result of Harter et al. [7], which showed that the coercive behavior of a superior had a negative effect on the work engagement of members. However, while coercion (*γ* = −0.08, *t* = −1.03) and reward (*γ* = −0.09, *t* = −1.19), which are part of coercive power, did not have a significant influence in the first-order construct analysis, legitimate power did have a statistically significant influence (*γ* = 0.14, *t* = 2.16). Therefore, it is determined that even if a person has more power than a counterpart, it is better to influence their work engagement by dealing with the counterpart based on a legal contract, rather than by using coercion or reward.

The hypothesis that coercive power influences OCB (H2) was not statistically significant (*γ* = 0.12, *t* = 0.28). This result is different from the research result of Ramaseshan et al. [13], which showed that coercive power decreases the satisfaction of workers and, long-term, decreases the loyalty toward the company. However, while coercion (*γ* = −0.09, *t* = −0.91) and reward (*γ* = 0.05, *t* = 0.02), which are components of coercive power, did not significantly influence OCB in the first-order construct analysis, legitimate power had a statistically significant influence (*γ* = 0.21, *t* = 2.37).

Therefore, even if a person has more power than a counterpart, forcing the counterpart to sacrifice will not be effective for improving performance. However, it will be better if OCB occurs in a legal/institutional relationship. 

The hypothesis that non-coercive power influences work engagement (H3) was statistically significant (*γ* = 0.53, *t* = 6.49). This result is the same as the research result of Christian et al. [18], which demonstrated that the expertise and reference of a superior increases the work engagement of the members. Additionally, information (*γ* = 0.28, *t* = 2.92), expert (*γ* = 0.31, *t* = 3.31), and reference (*γ* = 0.27, *t* = 3.16) power were shown to have a significant influence on work engagement in the first-order construct analysis. Consequentially, it is determined that non-coercive power based on building a continuous relationship helps improve work engagement.

The hypothesis that non-coercive power influences OCB (H4) was statistically significant (*γ* = 0.25, *t* = 2.57). This result is similar to that of Ilies et al. [28], which states that a good relationship built through non-coercive power has a positive effect on OCB. Information (*γ* = 0.21, *t* = 2.49), expert (*γ* = 0.24, *t* = 2.56), and reference (*γ* = 0.20, *t* = 2.26) power have a significant influence on OCB in the first-order construct analysis. Therefore, it is determined that non-coercive power based on common value creation helps improve OCB.

The hypothesis that work engagement influences OCB (H5) was statistically significant (*β* = 0.60, *t* = 9.04). This is similar to the research result of Rich and Lepine [6], which identified the positive relationship between work engagement and OCB. Consequentially, it is determined that a member with high work engagement exhibits OCB willingly, in addition to his/her own roles.

## 4. Discussion

This study aimed to analyze the effect of power type on work engagement and OCB. Here, power was classified into coercive power and non-coercive power. Accordingly, this study investigated the mutually beneficial causal relationship of classified powers and OCB through work engagement.

Therefore, the main contribution of this study is that the effect of power type on work engagement and OCB was analyzed empirically. Furthermore, this study performed a comparative analysis of conventional studies.

First, coercive power did not have an influence on work engagement and OCB. Analysis revealed that coercive power does not have any effect, even indirectly, on work engagement and OCB. However, in the first-order construct analysis, although coercion and reward had no significant influence, legitimate power had a significant effect. This means that coercive power does not have an influence on work engagement and OCB. Overall, coercive power does not have a desirable effect on the performance of an organization. Nevertheless, although coercion and reward had no influence, legal and institutional factors exhibited influence. For the improvement of organizational performance, it is necessary to endeavor to build a mutually dependent atmosphere based on reciprocity, rather than by using coercive power.

Second, non-coercive power showed similar effects on both work engagement and OCB. Furthermore, significant influences were seen on work engagement and OCB in both the first and second-order construct analysis. The analysis revealed that non-coercive power also has an indirect influence on OCB. These results are the same as those of Ilies et al. [28]. It is determined that a counterpart’s expert knowledge, information, and corporate culture increase the partnership. Therefore, non-coercive power factors in different companies should be widely accommodated to improve work engagement and OCB.

Third, work engagement had significant influence on OCB. Therefore, when work engagement increases, satisfaction will also increase naturally, and this will lead to OCB. Hence, it is proven that the work engagement of individuals is the most important factor for the performance improvement of an organization. This is consistent with the results of many conventional studies [6]. Based on these research results, company performance should be approached in a way such that the power type can lead to improvement of partnerships with others. Such a strategic approach will be able to increase the company performance the most effectively.

## 5. Conclusions

The contributions of this study, from the academic and practical perspectives, are as follows. First, practically, the relationship between power type and work engagement—which has been overlooked up until now—has been illuminated as one of importance. It is hoped that the power types that have practical influence will be further investigated, and plans will be made to develop and maintain better relationships within an organization.

Second, the causal relationship between power type and OCB in an organization was investigated, which has been lacking until now. The power types of coercive power or non-coercive power have both direct and indirect influences on the OCB in an organization, but since there is no previous study that has investigated this systematically, this study will be able to provide a new frame of understanding.

Third, and above all, by confirming the positive effect of work engagement on OCB through the results of this study, it is confirmed that work engagement is a very important concept for maintaining or improving the competitive advantage of an organization. Furthermore, based on the effect on OCB, it is determined that members with high work engagement can increase the effectiveness of an organization by creating social circumstances through discretionary behaviors, whereby team work, cooperation, and proper support is provided. Therefore, to increase the competitiveness of an organization, the leaders of the organization should first realize the importance of work engagement. Moreover, they should regularly measure and constantly strive to improve the work engagement of their members.

Although this study makes several positive academic and practical contributions, there are several limitations in terms of the research content and method. First, this study did not consider characteristics pertaining to the size and business sector of a company. Through segmentation of company size and business category, future studies should derive more significant results by increasing the representativeness of samples. There is also a need for a comparative analysis based on demographic variables.

Second, the effect of power type on OCB should be further investigated in a future study. Some studies have made partial attempts to do so, but an in-depth study is necessary. Tool development and verification research are required in order to include and measure a considerable number of variables.

Third, to accurately check the causal relationship between the variables used in this study, it is desirable to perform a longitudinal study conducted across several time points. This study was a cross-sectional design. Therefore, in a future study, data should be collected and analyzed over many time periods to clearly determine the causal relationship between variables.

## Figures and Tables

**Table 1:** Major literature on power and the issues covered.

References	Major Studies on Power
Hausman and Johnston [11]	Coercive Power/Non-Coercive Power
Maloni and Benton [15]	Mediated Power/Non-Mediated Power
Brown et al. [16]	Economic Power/Non-Economic Power

**Table 2:** Research constructs and operationalization.

Construct		Items	References
Coercive Power	Coercion	If I disagree with a proposal of a superior, I will be in an undesirable situation.	Harter et al. [7] Hausman and Johnston [11] Benton and Maloni [12] Maloni and Benton [15] Brown et al. [16]
		If I do not accept a request of a superior, I will be in an unfavorable situation.	
		If I do not accept a request of a superior, I receive a disadvantage.	
	Reward	If I do not accept a proposal of a superior, it will be difficult to receive an incentive.	
		If I do not accept a proposal of a superior, it will be difficult to receive economic benefits.	
		If I do not accept a proposal of a superior, it will be difficult to participate in a new project.	
	Legitimate	It is stated in the contract to accept proposals of a superior.	
		I have an obligation to accept the proposals of a superior.	
		The relationship is established such that I have to accept the proposals of a superior.	
		I have an obligation to accept the requests of a superior.	
Non-Coercive Power	Information	The superior can provide me with useful information.	Ramaseshan et al. [13] Bakker & Demerouti [17] Christian, et al. [18] Spagnoli et al. [21] Maslach et al. [22] Schaufeli and Bakker [23] Bakker and Demerouti [25] Sonnentag et al. [26]
		A work method of a superior can be helpful to me.	
		Because the decisions of a superior are rational, I reflect them in my work.	
		The superior provides me with reliable information.	
	Expert	The superior can provide me with helpful knowledge.	
		The superior can provide me with helpful experience.	
		The superior can provide me with helpful advice.	
		The superior can provide me with helpful decisions.	
	Reference	The value of a superior is a good example to follow.	
		The decision-making of a superior is a good example to follow.	
		The operation method of a superior is a good example to follow.	
		It is desirable to become like the superior.	
Work Engagement		I am full of energy when I work.	Harter et al. [7] Shin et al. [19] Navarro-Abal et al. [20] Tims et al. [27] Ilies et al. [28]
		My work motivates me to work hard.	
		I am passionate when performing my job.	
		My work is highly meaningful and valuable.	
		I am highly involved when performing my job.	
Organizational Citizenship Behavior (OCB)		I willingly spend time to help with the work-related problems of other employees.	Rich and Lepine [6] Kahn [24] Organ [29] Graham [30]
		I communicate effectively and work voluntarily.	
		Although it is not my duty, I strive to produce better job performance than what is stipulated.	
		I have loyalty and pride in the company.	

**Table 3:** Profiles of respondents.

Profiles of Respondents	Frequency	Percent (%)
Age of respondent		
30–40	89	48
40–50	66	36
Over 50	29	16
Gender of respondent		
Male	116	63
Female	68	37
Job tenure of respondent		
1–5	69	37
5–10	62	34
Over 10	53	29
Title of respondent		
Assistant manager	62	34
Manager	57	31
General manager	42	23
Executive director	23	11
Industry		
Manufacturing/engineering	59	32
Services and utilities	42	23
Transportation and logistics	45	24
Retailing and wholesale	38	21
Number of employees		
Less than 1000	52	28
1001–5000	79	43
More than 5000	53	29

**Table 4:** Results of confirmatory factor analysis (each item is measured with a seven-point Likert type scale)

Item	Coercion	OCB	Expert	Reference	Legitimate	Work Engagement	Information	Reward	Mean (S.D.)
CO1	0.776	−0.101	0.110	0.156	0.257	−0.065	−0.023	−0.252	4.30 (1.52)
CO2	0.828	−0.126	0.102	0.032	0.279	−0.031	−0.120	−0.274	
CO3	0.861	−0.068	0.051	0.097	0.123	−0.079	−0.034	−0.242	
RE1	0.402	0.003	−0.087	0.082	0.193	−0.020	0.065	0.809	3.88 (1.39)
RE2	0.392	−0.001	−0.073	0.084	0.154	−0.076	0.127	0.832	
RE3	0.219	−0.006	−0.003	0.000	0.134	−0.072	0.081	0.859	
LE1	0.214	0.074	0.052	0.071	0.835	−0.012	0.069	0.031	3.80 (1.57)
LE2	0.223	0.157	0.125	0.122	0.873	0.049	0.064	0.060	
LE3	0.262	0.042	0.119	0.069	0.863	0.132	−0.088	−0.056	
LE4	0.158	0.132	0.042	0.086	0.901	0.056	0.051	0.005	
IN1	−0.164	−0.062	0.470	0.139	0.023	0.226	0.566	−0.220	4.72 (0.93)
IN2	−0.017	0.126	0.258	0.271	0.072	0.102	0.747	0.126	
IN3	0.247	0.242	0.098	0.451	0.087	0.119	0.534	0.385	
IN4	0.204	0.012	0.287	0.381	−0.010	0.249	0.604	−0.032	
EX1	0.075	0.074	0.890	0.065	0.124	0.031	0.105	−0.053	5.11 (1.06)
EX2	−0.002	0.119	0.915	0.175	0.109	0.098	0.089	0.035	
EX3	0.037	0.123	0.901	0.176	0.034	0.062	0.163	−0.005	
EX4	0.006	0.217	0.837	0.181	0.065	0.111	0.123	0.038	
RF1	0.130	0.163	0.189	0.827	0.067	0.085	0.160	0.019	4.47 (1.10)
RF2	0.098	0.228	0.102	0.835	0.170	0.151	0.189	0.005	
RF3	0.027	0.142	0.199	0.871	0.061	0.191	0.056	0.088	
RF4	0.086	0.016	0.148	0.830	0.094	0.145	0.219	−0.106	
WE1	−0.078	0.384	−0.041	0.289	−0.034	0.594	0.414	−0.235	4.74 (1.01)
WE2	−0.062	0.402	0.041	0.198	0.069	0.643	0.196	−0.162	
WE3	−0.089	0.152	0.162	0.098	0.134	0.859	0.104	0.110	
WE4	−0.125	0.465	0.134	0.282	0.039	0.697	0.009	0.091	
WE5	−0.087	0.447	0.121	0.247	0.042	0.776	0.237	−0.076	
OCB1	−0.075	0.817	0.084	0.087	0.037	0.282	−0.011	0.115	4.36 (1.40)
OCB2	0.016	0.873	0.192	0.124	0.152	0.077	0.148	−0.055	
OCB3	−0.154	0.832	0.125	0.017	0.083	0.334	−0.048	0.012	
OCB4	−0.004	0.814	0.128	0.247	0.160	0.148	0.080	0.025	

CO: Coercion, RE: Reward, LE: Legitimate, IN: Information, EX: Expert, RF: Reference, WE: Work Engagement, OCB: Organizational Citizenship Behaviors, the shaded numbers (≥0.50).

**Table 5:** Results of convergent validity.

Measures	AVE	CR	Cronbach α
Coercion	0.604	0.846	0.914
Reward	0.642	0.861	0.939
Legitimate	0.581	0.852	0.932
Information	0.622	0.826	0.795
Expert	0.561	0.768	0946
Reference	0.619	0.861	0.926
Work Engagement	0.553	0.838	0.916
Organizational Citizenship Behaviors	0.699	0.841	0.916

**Table 6:** Results of discriminant validity.

Construct	Coercion	Reward	Legitimate	Information	Expert	Reference	Work Engagement	OCB
Coercion	0.777							
Reward	0.690 **	0.801						
Legitimate	0.427 **	0.374 **	0.762					
Information	0.099	0.139	0.195 **	0.789				
Expert	0.118	0.012	0.222 **	0.514 **	0.749			
Reference	0.136	0.162	0.258 **	0.633 **	0.385 **	0.787		
Work Engagement	0.138	0.115	0.171 *	0.498 **	0.302 **	0.481 **	0.744	
OCB	0.118	0.040	0.244 **	0.307 **	0.321 **	0.332 **	0.669 **	0.836

The shaded numbers in the diagonal row are square roots of the AVE, * significant at α = 0.05 ** significant at α = 0.01.

**Table 7:** Variance inflation factor (VIF) and tolerance.

Construct	Tolerance	VIF	Construct	Tolerance	VIF
Coercive Power	0.913	1.095	Non-Coercive Power	0.671	1.490
Work Engagement	0.709	1.410	Dependent Variable: Organizational Citizenship Behaviors		

**Table 8:** Fit statistics for validating the measurement model.

Recommended Value		Measurement Model
Fit statistic	*X*^2^/DF (≤3.000)	2.670
	GFI (≥0.900)	0.912
	RMSR (≤0.050)	0.041
	RMSEA (≤0.080)	0.039
	AGFI (≥0.800)	0.813
	CFI (≥0.900)	0.892
	TLI (≥0.900)	0.918
	PGFI (≥0.600)	0.634

**Table 9:** Coefficients of direct, indirect, and total impacts.

Construct		Work Engagement	OCB
Coercive Power	Direct Effect	0.15	0.12
	Indirect Effect	-	0.01
	Total Effect	0.15	0.13
Non-Coercive Power	Direct Effect	0.53 **	0.25 **
	Indirect Effect	-	0.20 **
	Total Effect	0.53 **	0.45 **
Work Engagement	Direct Effect		0.60 **
	Indirect Effect		-
	Total Effect		0.60 **

* Significant at α = 0.05; ** significant at α = 0.01.
